# Balloon-assisted technique for endoscopic en bloc retrieval of a large gastric gastrointestinal stromal tumor specimen

**DOI:** 10.1055/a-2061-7227

**Published:** 2023-04-17

**Authors:** Suhua Wu, Song He, Zhechuan Mei, Feng Xu, Xiaodong Guo, Yingchi Zhang, Chao Deng

**Affiliations:** Department of Gastroenterology, The Second Affiliated Hospital of Chongqing Medical University, Chongqing, China

A 64-year-old woman underwent gastroscopy and abdominal computed tomography, which revealed a 4.0 × 3.5 cm gastric gastrointestinal stromal tumor (GIST) in the anterior wall of the stomach ( Fig. 1 a ). After full evaluation, an endoscopic full-thickness resection technique was used to achieve complete resection ( Fig. 1 b ). It is very important to retrieve the complete specimen to allow for subsequent pathological evaluation; however, retrieval is difficult for specimen larger than 3.5 cm. Wu et al. successfully applied a balloon-assisted technique for removal of large esophageal foreign bodies 1 . Therefore, we applied this technique for endoscopic en bloc retrieval of a large gastric GIST specimen ( Video 1 ).

**Fig. 1:**
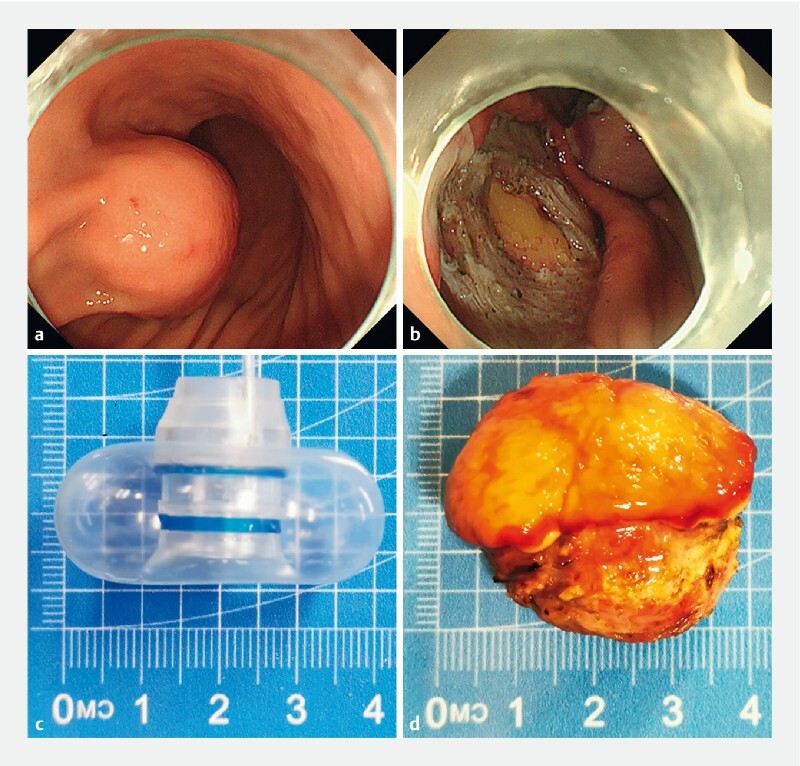
Endoscopic en bloc retrieval of a large gastric gastrointestinal stromal tumor (GIST) specimen using a transparent cap with a balloon. **a** A 4.0 × 3.5 cm gastric GIST was located in the anterior wall of the stomach. **b** The omentum was visible on the wound after complete resection. **c** A transparent cap with a balloon, which could be expanded to a maximum outer diameter of 4.0 cm, was used to assist retrieval. **d** The specimen had a volume of 4.0 × 3.5 × 3.5 cm and was retrieved en bloc.

**Video 1** The balloon-assisted technique for endoscopic en bloc retrieval of a large gastric gastrointestinal stromal tumor specimen.


After the wound had been completely sutured by clips and endoloop, attempts were made to retrieve the specimen with a snare, but passage through the cardia was impossible owing to the angle between the fundus of the stomach and the cardia. A transparent cap with a balloon, which could be expanded to a maximum outer diameter of 4.0 cm (Vedkang, Changzhou, China) was connected to the front end of the endoscope ( Fig. 1 c ). With the balloon expanded to 4.0 cm, the snare could easily drag the specimen into the esophagus. At the entrance of the esophagus, the resistance was greater, so the balloon size was reduced and the dragging force of the snare was increased to hold the transparent cap in place and prevent the balloon from slipping. Finally, the specimen was successfully removed in its entirety, and no bleeding or perforation was observed in the gastric cardia or esophageal mucosa. The specimen volume was 4.0 × 3.5 × 3.5 cm ( Fig. 1 d ). Histological examination showed a GIST with low risk for disease progression and negative vertical and horizontal margins. The patient had no complications and was discharged successfully 1 week after surgery.

Endoscopy_UCTN_Code_TTT_1AO_2AC

## Notes

Endoscopy E-Videos

https://eref.thieme.de/e-videos
*E-Videos* is an open access online section of the journal *Endoscopy* , reporting on interesting cases and new techniques in gastroenterological endoscopy. All papers include a high-quality video and are published with a Creative Commons CC-BY license. Endoscopy E-Videos qualify for HINARI discounts and waivers and eligibility is automatically checked during the submission process. We grant 100% waivers to articles whose corresponding authors are based in Group A countries and 50% waivers to those who are based in Group B countries as classified by Research4Life (see: https://www.research4life.org/access/eligibility/ ). This section has its own submission website at https://mc.manuscriptcentral.com/e-videos
